# A Multifeature Learning and Fusion Network for Facial Age Estimation

**DOI:** 10.3390/s21134597

**Published:** 2021-07-05

**Authors:** Yulan Deng, Shaohua Teng, Lunke Fei, Wei Zhang, Imad Rida

**Affiliations:** 1 School of Computer Science and Technology, Guangdong University of Technology, Guangzhou 510006, China; 13724753747@163.com (Y.D.); shteng@gdut.edu.cn (S.T.); weizhang@gdut.edu.cn (W.Z.); 2 Centre de Recherches de Royallieu, Université de Technologie de Compiègne, 76800 Compiègne, France; imad.rida@utc.fr

**Keywords:** age estimation, multifeature learning, feature fusion, regression-ranking estimator

## Abstract

Age estimation from face images has attracted much attention due to its favorable and many real-world applications such as video surveillance and social networking. However, most existing studies usually learn a single kind of age feature and ignore other appearance features such as gender and race, which have a great influence on the age pattern. In this paper, we proposed a compact multifeature learning and fusion method for age estimation. Specifically, we first used three subnetworks to learn gender, race, and age information. Then, we fused these complementary features to further form more robust features for age estimation. Finally, we engineered a regression-ranking age-feature estimator to convert the fusion features into the exact age numbers. Experimental results on three benchmark databases demonstrated the effectiveness and efficiency of the proposed method on facial age estimation in comparison to previous state-of-the-art methods. Moreover, compared with previous state-of-the-art methods, our model was more compact with only a 20 MB memory overhead and is suitable for deployment on mobile or embedded devices for age estimation.

## 1. Introduction

Age estimation is performed to identify a human’s age from face images, which has broad application scenarios in public areas. For example, when police search for criminals through video surveillance, they can quickly narrow the search range by using age estimation. So far, there have been a variety of methods proposed for age estimation [1,2]. Most existing facial age estimation systems usually consist of two key stages: age-feature learning and age-feature estimator. Age-feature learning aims to learn more age features from face images to make age information separable. Traditional age-feature-learning methods are based on hand-crafted features, such as the Local Binary Pattern (LBP) [3], the Histogram of Oriented Gradients (HOG) [4], and Biologically Inspired Features (BIF) [5]. However, these hand-crafted-based features require strong prior knowledge to engineer them by hand [6]. To address this limitation, deep-learning-based techniques have been proposed and have shown great success in age-feature learning in recent years [7,8]. For example, Yi et al. [7] proposed a multiscale framework to learn deep age features for age estimation. Wang et al. [8] developed an end-to-end learning approach to learn robust age features and achieved very competitive performance compared with hand-crafted-based methods. Due to this, the recent age-feature-learning studies mainly focus on deep-learning networks such as VGG-16 [9], AlexNet [10], and MobileNet [11].

On the one hand, although very competitive performance has been achieved, most state-of-the-art deep-learning networks are often bulky with more than 300 MB and not suitable to be adapted to platforms with limited memory such as mobile and embedded devices [12,13]. Thus, some studies are focused on designing compact deep-learning networks for age estimation, so that these deep-learning models can be embedded in small memory devices. For example, Yang et al. [12] adopted a two-stream CNN model to estimate age, and the model consumed around 1 MB. Niu et al. [14] proposed ORCNN with only 1.7 MB of consumption. These compact models sacrifice some performance for a smaller memory space.

**Figure 1:**
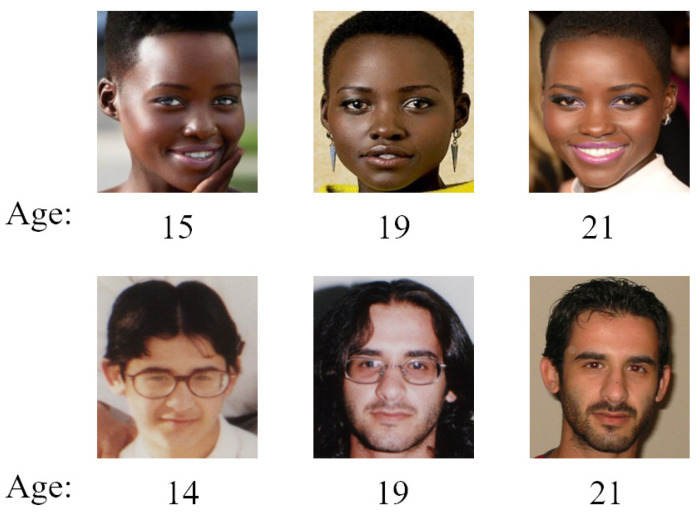
Gender and race have some effects on aging patterns. For example, we can see that women do not have beards at any age and men grow beards as they become older.

On the other hand, it is widely observed that the information of race and gender is highly correlated with age features, which also exist in the form of pixels in facial images. For example, females usually look younger than males of similar ages when they are young and look older than males when they are old. Men have less of a beard or none when they are young, but they have more of a beard when they are old. On the contrary, no matter whether young or old, women never have a beard, as shown in Figure 1. However, most age-feature-learning approaches focus on learning a single kind of age feature and ignore other appearance features such as gender and race, which have a great influence on the age pattern [15]. Inspired by this, we utilized three compact subnetworks to learn multiple features from the same input image and fused these features to form more discriminative and robust features for age estimation.

For the age-feature estimator, it mainly converts the extracted age features into exact age numbers. In general, the age estimator can be considered as a classifier or a regressor. Representative classifier-based methods include Support Vector Machines (SVMs) [16], Random Forests (RFs) [17], and k Nearest Neighbors (k-NNs) [18]. Classifier-based methods equally treat different ages as independent classes, which ignores the inherent relationship of age labels. Therefore, the costs of classifying a young subject as a middle-aged subject and an old subject are the same. Due to this, many regression-based methods [1,9] were proposed to make use of the continuity of age labels. For example, Agustsson et al. [19] proposed a nonlinear regression network for age estimation. Geng et al. [20] proposed a CPNN algorithm to learn age-regression distributions. The regressor-based method oversimplifies the aging pattern to a linear model. However, the facial-aging pattern is generally a nonlinear problem and an extremely complex process, affected by many factors [21]. To avoid the problem of over linearization, some ranking-based methods have been proposed for age estimation [22,23], and these approaches treat the age label as an ordinal sequence. For example, Zhang et al. [24] proposed a paradigm for mapping multiple age comparisons into an age-distribution posterior for age estimation. Chen et al. [23] proposed a ranking-CNN model with a series of basic networks, and their binary outputs were aggregated for the final age prediction. For ranking-based methods, features are learned independently in each age group to depict different aging patterns, which avoids the overlinearization problem of the regression-based model. However, most ranking-based methods are built on complex networks or ensembles of networks. These models are often bulky and not suitable to be adapted to platforms with limited memory and computation resources such as mobile and embedded devices.

In this paper, we proposed a new age-feature descriptor by exploring multiple types of appearance features and engineered a regression-ranking estimator for robust age estimation. Specifically, we first used three compact subnetworks to learn gender, race, and age information from the same input image. Then, we fused these complementary features to further form more discriminative and robust features. Finally, we used a regression-ranking-age estimator to predict the final age number based on the fusion features. Compared to the approaches based on ranking technology or regression technology, our proposed method could better utilize the order and continuity of age labels. Moreover, our model was more compact with only 20 MB of memory overhead. The experimental results showed the effectiveness and efficiency of the proposed method on facial-age estimation in comparison with previous state-of-the-art methods.

The main contributions of this paper can be summarized as follows:

- We proposed a compact multifeature-learning network for age estimation by learning and fusing the gender, race, and age information. By integrating these complementary features, more discriminative and robust age features could be obtained in the final feature descriptor;
- We engineered a regression-ranking estimator to convert the fusion features into exact age numbers, which could simultaneously make use of the continuity and the order of the age labels;
- We conducted extensive experiments on three widely used databases. The experimental results clearly showed that our proposed method could achieve a higher accuracy for age estimation than most state-of-the-art age-estimation methods.

The remainder of this paper is organized as follows. Section 2 reviews the related work. Section 3 shows the details of our proposed method. The experiments and results are illustrated in Section 4. Finally, we draw conclusions in Section 5.

## 2. Related Work

In this section, we briefly review three related works including age-feature learning, multifeature learning and fusion, and age-feature estimator.

### 2.1. Age-Feature Learning

Human age estimation has been studied extensively for over 20 years. One of the earliest age-estimation models can be traced back to [25], which extracted texture and appearance features from a small number of training samples. At that time, most age-estimation methods were based on the singlelocal features due to the limited number of training samples. Thus, most age-estimation methods were based on the single local features. For example, Guo et al. [5] extracted the biologically inspired features from facial images and then performed statistical learning for human-age estimation. Gao et al. [26] proposed a fuzzy LDA method using Gabor features for coarse-age classification. Gunay et al. [18] extracted local binary patterns for face descriptions, which were the fundamental properties of the local-image-texture and effective-texture features. Recently, deep learning has gained much success on age-feature learning. For example, Levi et al. [27] proposed a simple convolutional net architecture for age estimation and validated the performance of the deep-learning network on unconstrained facial images. In recent years, with the development of GPUs, CNN models with deep architectures have achieved breakthroughs on pattern recognition, and more and more age estimation models are based on CNNs. For example, Abdulnabi et al. [28] proposed a joint deep-learning network to perform multitask recognition including for gender, age, and skin. Huerta et al. [29] proposed a deep-learning scheme for accurate age estimation based on the fusion features. In addition, to make the CNN model more effective, some new components were introduced such as Exponential Line Units (ELU) and Batch Normalization (BN). The ELU makes the model converge faster in training, and BN makes the model pay more attention to the global features instead of the local features, which make the generalization ability of the model stronger [30]. Based on the above results, we decided to integrate the CNN with the ELU and BN to improve the prediction accuracy. The details are presented in Section 3.

### 2.2. Multifeature Learning and Fusion

In the past, most age-estimation methods only considered a single age feature. Specifically, the target of these single-feature-based models was only the age class. Thus, single-feature-based models directly learn the abstract age features. However, the age feature is a complex feature that is affected by many factors such as gender and race. To learn the more discriminative and robust age features, there have been many multifeature-based methods proposed for age estimation [6,15], which extracted multiple types of information as the age-feature descriptors. For example, Antipovet et al. [31] presented a deep-learning model for age estimation by fusing the general and child-specialized features. Yaman et al. [32] proposed a multimodal age-estimation method by combining the ear and profile face. Yang et al. [12] utilized a two-stream model to learn and integrate different age features for age estimation. The extensive experimental result showed that the multifeature method obtained more discriminative and robust age features and achieved a better performance compared with other single-feature-based methods.

After obtaining multiple features, the fusion technology of multiple features also has an important influence on age estimation. Generally, there are three ways of feature fusion including intensity fusion, spatial fusion, and channel fusion [32]. In intensity fusion, the pixel value of each feature map is weighted and added. Therefore, multiple feature maps will eventually be integrated into one feature map. In spatial fusion, multiple feature maps are concatenated side-by-side. For example, the left half of the concatenated map is the gender map, and the middle part is the age map, while the right half includes the race map. In channel fusion, each feature map is concatenated along the channels. For example, the dimensions of gender, race, and age feature map are [224, 224, 1], after channel fusion, and the fusion feature map is [224, 224, 3]. Among these three ways, channel fusion is more suitable for the fusion of multiple features learned from a single image [12,32]. Different from most existing multifeature-learning methods that extracted multiple types of features from multimodel images [31,32], we simultaneously learned and fused multiple types from features from single-model face images for age estimation. Therefore, we decided to explore multifeature learning and channel fusion for age estimation.

### 2.3. Age-Feature Estimator

Given the age feature representation, the age estimator mainly converts the age features into exact age numbers. In the past, the age estimator was modeled as a classifier. For example, Zheng et al. [33] proposed a PCANet to estimate human age based on the softmax loss. Soumaya et al. [34] presented an autoencoder network to classify the age label based on unsupervised learning. Note the fact that the age label is a continuous value rather than a set of discrete classes [35]. Thus, to make use of the continuity of age labels, regression-based methods were proposed for age estimation in recent years. For example, Rothe et al. [1] first used the expected value on the softmax probabilities and then calculated the regression age. Zhang et al. [36] presented the age representation as a distribution over two discrete adjacent bins. To better exploit the ordinal relationship among age labels, a few ranking-based methods were proposed recently. For example, Xie et al. [22] proposed an ordinal-ensemble-learning network for age estimation. Chen et al. [23] proposed a ranking-CNN model that contained a series of basic CNNs, which converted the age-estimation problem into multiple binary classification tasks. In this work, we simultaneously used regression-and-ranking age-prediction schemes to engineer the age-feature estimator.

## 3. Proposed Method

In this section, we first present the overall framework of the proposed method. Then, we illustrate the multifeature learning and the regression-ranking estimator of the proposed method.

### 3.1. The Framework of the Proposed Method

On the one hand, the human age pattern is complicated and is easily affected by many factors, such as identity, gender, race, and extrinsic factors. However, most models focus on single-age-feature learning and ignore gender, race, and other age-related features. Due to this, we aimed to learn more robust age features by exploring the potential gender and race information from the same original images. On the other hand, most deep-learning-based age-estimation methods are built on complex networks or ensembles of networks, which require a large sample and memory to train the network. Thus, they are not suitable to be adapted to platforms with limited memory and computation resources such as mobile and embedded devices. To reduce the model size without sacrificing much accuracy, we first broke down the complex age problem into three simple feature-learning tasks. Then, we fused these age-related features in the fully connected layer and fed them into the age-feature estimator to predict the final age.

**Figure 2:**
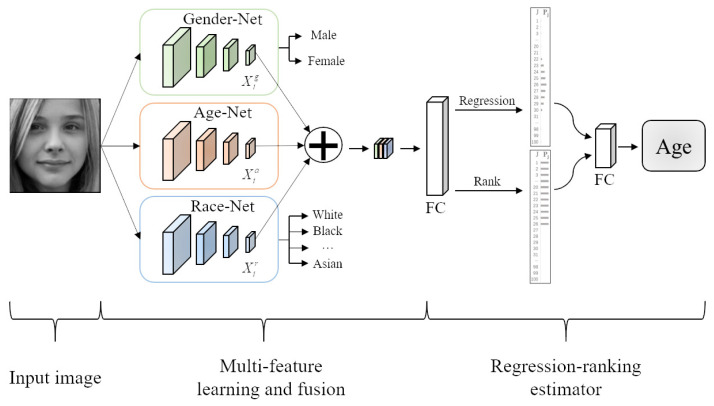
An overview of the proposed method. We first use three subnetworks to learn the gender, age, and race features from the same image. Then, we fuse these features and input the fusion features into the regression-rank estimator to predict the final age.

Figure 2 shows the basic idea of the proposed method, which mainly consisted of two parts: multifeature learning and fusion and regression-ranking estimator. In the first part, we firstly utilized Gender-Net, Age-Net, and Race-Net to learn multiple types of features from the same input image. Then, we fused the features in the full connection layer such that more discriminative and robust features could be obtained. In the second part, to simultaneously utilize the continuity and the order of age labels, we engineered the regression-ranking estimator to predict the final age based on the fusion features. In the following, we present the detailed procedures of the two parts of the proposed method.

### 3.2. Multiple Types of Features’ Learning and Fusion

To learn more discriminative and robust features, we first employed three subnetworks to learn the gender, age, and race information from the same input image, as shown in Figure 2. Each network was composed of 4 convolution blocks, and each convolution block included a convolutional layer, a nonlinear activation, a batch normalization, and a pooling layer. For the convolutional layer, it mainly learned the target feature from the previous feature map and output a new feature map. Specifically, we respectively used 32 and 64 kernels with a size of 5 × 5, a stride of 1 pixel, and 0 padding for the first and second layers to learn the coarse features. Then, we used the following two layers to learn the subtle features, which respectively used 64 and 128 kernels with a size of 3 × 3, a stride of 1, and 1padding. The outputs of the convolutions added an element-wise nonlinear activation function to normalize the front results. Without loss of generality, we used the ELU function as the nonlinear activation function to deactivate the output of the convolutional layer. For the batch normalization, it not only aimed to improve the learning of the local features, but also to improve the learning of the overall features [37]. To achieve the target, we used the subtractive and divisive normalization operations [35,38] to normalize each feature map. For the pooling layer, it mainly converted the feature map into a more representative feature-representation map with a smaller size scale. For example, after the pooling layer, the feature map [64, 64] was transformed into a feature-representation map [32, 32]. Although the size of the feature-representation map was smaller, it contained the main feature information, which was helpful to save computing resources and improve the accuracy of the feature recognition. In our experiments, we used the maximum pooling operation to generate the feature-representation map.

These three subnetworks took the same image with the gender, age, and race labels as the input. For example, $I_{i}=\;\left\{male,25,white\right\}$ represents a 25-year-old white man. For the gender and race attributes, we used one-hot coding [39] to encode these attributes, in which only one feature map was filled with one and others were filled with zero. For example, the output of the Gender-Net [1, 0] represents a male and [0, 1] a female. Through the learning procedure of the three subnetworks, the high-level-feature maps of gender, age, and race could be learning and recorded in the convolution layer. Then, we fused these feature maps and fed them into the full connection layer, so that more discriminative and robust age features could be obtained in the final feature descriptor. When the input features had more information, the age estimator would be more flexibility at predicting the age of people with different genders and races. Thus, the age-feature estimator could achieve a better generalization performance.

For the feature map fusion, we aimed to obtain an enhanced feature that was beneficial to the age-feature estimator. Specifically, let $X_{i}^{g},X_{i}^{a},X_{i}^{r}\in R^{1\times w\times h}$ be the gender, age, and race feature maps, respectively, and ⊕ denote the channel-connection operation; $new\;feature\;map=\left[\left[X_{i}^{g}\right],\left[X_{i}^{a}\right],\left[X_{i}^{r}\right]\right]\in R^{3\times w\times h}$ is formed and input into the regression-ranking-age estimator to predict the final age. In other words, we first broke down the complex task of age estimation into three simple subtasks. Then we separately learned the gender-specific, race-specific, and age-specific features. After that, we fused these complementary features to form the more discriminative and robust age features. Finally, we fed the fusion features into the age-feature estimator to predict the final age.

### 3.3. Regression and Ranking Estimator

Given the age-feature representation, the age estimator aimed to predict the age of the face in the image. Generally, the age estimator could be modeled as a regression model or a ranking model. Different from single-regression-based methods or single-ranking-based methods, we first engineered a regression-age estimator and a ranking-age estimator to simultaneously make use of the continuity and the order of the age labels. Then, we balanced the effects of continuity and the order of the age label and made a good tradeoff between them. Specifically, we used two fully connected layers to engineer the regression-age estimator and ranking-age estimator, respectively. The output of the regression-age estimator was the age probability $\left\{p_{i}\in \left[0,1\right]|i=1,2,\ldots ,n\right\}$, and the output of the age-ranking estimator was the age ranking $\left\{p_{j}=0\;\mathrm{or}\;1|j=1,2,\ldots ,n\right\}$. For the regression age, we computed the expected value as the age number, as follows:

(1) $$regression\;age=\sum_{i=1}^{n}J_{i}\times P_{i}=\left[1,2,\ldots ,a_{n}\right]\;\times \;{\left[p_{1},p_{2},\ldots ,p_{n}\right]}^{T},$$

where $J_{i}$ is the age label and $P_{i}$ denotes the probability that the input image belongs to the age of $J_{i}$. For the ranking age, we first denoted the face image as $\left(I_{i},Y_{i}\right)$, where $I_{i}$ is the *i*-th input image and $Y_{i}\in \left\{1,\ldots ,1,y,0,\ldots ,0\right\}$ is the corresponding ranking-age label, which means that the age of the *i*-th input image is *y*. The age number can be computed as:

(2) $$ranking\;age=\sum_{j=1}^{n}\left[p_{j}>\mu \right],$$

where μ is the threshold value and $\left[\cdot \right]$ represents the true and false check operator, which outputs 1 when the internal condition is true, and 0 otherwise. It can be seen that age estimation was turned into a ranking problem by minimizing the binary ranking errors.

Age regression and age ranking respectively estimate the age from the age-continuity and age-order properties, such that they are complementary for the final age number estimation. After age regression and age ranking, we further forwarded them to a fully connected layer to estimate the final age as follows:

(3) $$age=\alpha_{1}\sum_{i=1}^{n}J_{i}*P_{i}+\alpha_{2}\sum_{i=1}^{n}\left[p_{j}>0\right],$$

where $\alpha_{1}$ and $\alpha_{2}$ are two parameters of the final fully connected layer to balance the effects of the continuity and order of the age label and make a good tradeoff between them.

## 4. Experimental Setup and Results

In this section, we conducted age-estimation experiments on the widely used MORPH2 [40], FG-NET [41], and LAP [42] datasets. Our method was implemented within the PyTorch framework. The parameters of the proposed networks were all initialized with the Xavier initialization, and Adam was used as the optimizer. The learning rate and batch size were empirically set to 0.001 and 16, respectively, and the RLRP algorithm was used to automatically adjust the learning rate. The experiments were performed on the same machine with a GTX 2060s graphics card (including 2176 CUDA cores), a i5-9600KF CPU, and a 32 GB RAM.

### 4.1. Datasets and Preprocessing

MORPH2 is the most popular dataset for age estimation, which contains 55,134 face images of 13,617 subjects with the age ranging from 16 to 77. Among them, there are 77% face images from Africa, 19% from America, and the rest from Asia. FG-NET is a very recent database used for age estimation, which contains one-thousand two face images of eight-two individuals with the age ranging from zero to sixty-nine. All of them are from Europe and America. LAP contains 4691 face images, of which approximately 81% of the face images are from America, 11% are from Africa, and the rest are from Asia. Table 1 tabulates the information of three databases, as well as their experimental settings. The age distributions of the MORPH2, FG-NET, and LAP datasets are shown in Table 2. In addition, we employed the IMDB-WIKI database of 523,051 images to pretrain our proposed network.

As shown in Table 1, the training images of the FG-NET, MORPH II, and LAP databases are extremely insufficient. For example, FG-NET contains no more than eight-hundred training images, which is far from enough to train a deep-learning network. Although MORPH2 has 44,000 training samples, it is not enough for a deep model to reach convergence [10,43]. Therefore, increasing the training samples was necessary to improve the performance. To enlarge the sample sets, we first flipped each image to obtain two mirror-symmetric samples and then rotated them by ±5° and ±10°. Moreover, we added Gaussian white noise with variances of 0.001, 0.005, 0.01, 0.015, and 0.02 on the original and the synthetic samples, so that each image was finally extended to 40 samples.

**Figure 3:**
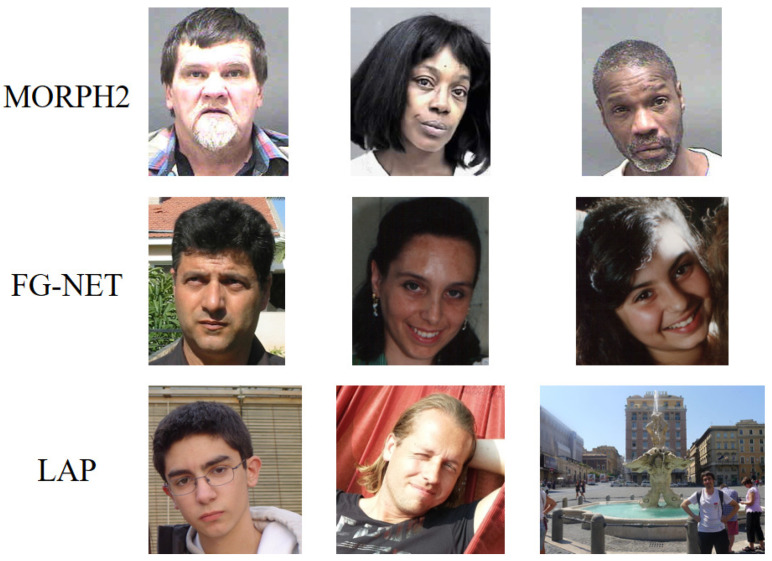
Some samples of the MORPH2, FG-NET, and LAP databases. These is much noise in the original image such as illumination variations and different postures. For example, the face posture in the first line is different, and the strength of illumination in the second row of images is different.

**Figure 4:**
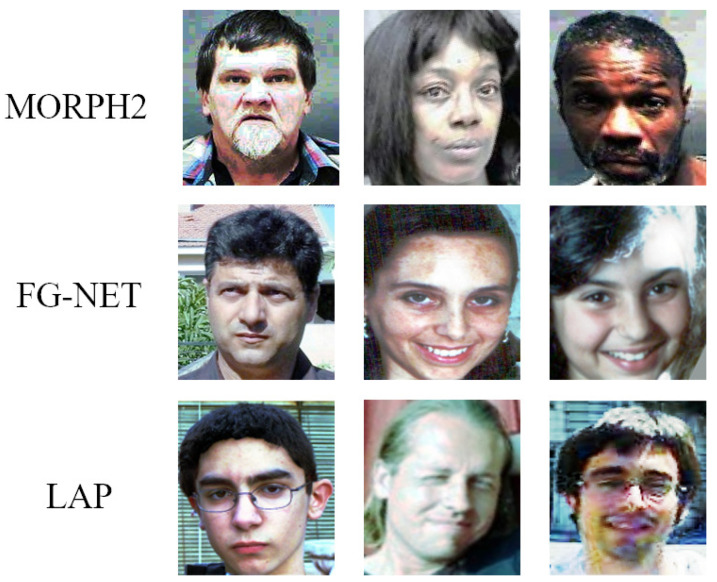
Some of the processed images from the MORPH2, FG-NET, and LAP databases.

Figure 3 shows some samples from MORPH2, FG-NET and LAP. We can see that there was much noise in the facial images such as illumination variations and different postures. As pointed out by [44], illumination compensation and normalization have an important impact on facial attribute analysis. Therefore, we used DT-CWT [45] to normalize the illumination in the experiments. After that, all face images were first processed by a face detector [46], and a few nonface images were removed. Then, we used AAM [47] to align all faces, according to the eyes’ center and the upper lip. Finally, all face images were cropped into a size of 224 × 224 and then fed into the network. Some of the processed images are shown in Figure 4. From Figure 4, we can see that these images were well normalized in terms of illumination and posture. For example, the illumination of the images in the middle row of Figure 3 was relatively dark. After the illumination normalization, the illumination of these images increased. The face of the third row of Figure 3 was detected, and the pose was normalized according to the eyes’ center and the upper lip.

### 4.2. Evaluation Metrics

For the evaluation of the age estimation models, the Mean Absolute Error (*MAE*) is the most commonly used evaluation indicator, which represents the absolute average error between the predicted age and the true age. *MAE* is calculated as follows:

(4) $$MAE=\frac{1}{n}\sum_{i=1}^{n}\left|{y_{i}}^{\prime }-y_{i}\right|,$$

where ${y_{i}}^{\prime }$ and $y_{i}$ represent the predicted age and the true age, respectively, and *n* is the total number of test samples. Obviously, a lower *MAE* value indicates a better performance of the model. On the contrary, a higher *MAE* value means a worse performance of the model.

The Cumulative Score (*CS*) is another important evaluation metric of the age-estimation model. We set the cumulative prediction accuracy as the error θ, and $CS\left(\theta \right)$ can be calculated as follow:

(5) $$CS\left(\theta \right)=\frac{N_{e<\theta }}{n}\times 100\%,$$

where $N_{e<\theta }$ denotes the total number of test samples on which the error of age prediction was less than θ. For example, we only counted the number of samples whose predicted age error was less than five if θ was set as five. Obviously, a higher $CS\left(\theta \right)$ value means a better performance of the model. On the contrary, a lower $CS\left(\theta \right)$ value means a worse performance of the model.

### 4.3. Comparisons with the State-of-the-Art

To validate the performance of our proposed multifeature-learning-and-fusion method, we compared our proposed method with different feature-based methods. The competing methods can be roughly categorized into two groups, single-feature-based methods and multifeature-based methods. The single-feature-based methods included DEX [1] and MSFCL [6]. These single-feature-based methods only consider the age feature and ignore other age-related features such as race and gender. The multifeature-based methods included DCP [35] and CNN2ELM [10]. DCP focuses on exploring the influence of race factors on age estimation. CNN2ELM extracts the age, race, and gender features from multimodel images and fuses them for age estimation. Different from CNN2ELM, we simultaneously learned and fused multiple types if features from the single-model face images for age estimation. For each dataset, eighty percent of the samples were used for training and the rest for testing. All models were pretrained with the IMDB-WIKI dataset. Table 3 tabulates the *MAE*s of different feature-based methods on the three databases.

We can see that multifeature-based methods had better performance on age estimation than single-feature-based methods. This is because human age estimation is a complicated process that is easily affected by race and gender. The multifeature-based method learned different age-related features and fused them to form the more discriminative and robust age features. In addition, our proposed method consistently outperformed the two multifeature-based methods by achieving lower *MAE*s. This was because our proposed method simultaneously learned and fused multiple types of features from the same input image, which could provide more relevant age-related features. Moreover, we engineered a regression-ranking age-feature estimator, which could make better use of the continuity and order of the age label.

To validate the performance of our proposed regression-and-ranking-estimator method on age estimation, we compared it with four representative age-estimation methods including GA-DFL [9], DOEL [22], C3AE [36], and CNN2ELM [15]. To make a fair comparison with the state-of-the-art methods, we adopted the same experimental setting as the work in [1]. We first used the training samples to train our method. Then, we used the testing samples to validate the performance of our method and calculated the Mean Absolute Error (*MAE*) [1]. Table 4 tabulates the *MAE*s of the different methods on the three databases.

It can be seen that our proposed method consistently outperformed the four compared methods by achieving obviously lower *MAE*s. This was because the proposed method adaptively fused the regression-and-ranking-age estimator, which can fully utilize the continuity and the ordinal relationship of the age labels. Another possible reason was that our proposed method first used three subnetworks to learn the gender, race, and age information and then fused these complementary features, such that more discriminative and robust age features could be obtained in the final feature descriptor, which improved the performance of our proposed method on age estimation.

To better validate the effectiveness and efficiency of the proposed method, we compared it with a set of state-of-the-art deep-learning-based age-estimation methods. The competing methods can be roughly categorized into two groups, bulky models and compact models, based on their model sizes. The bulky models included DEX [1] and RankingCNN [23]. These bulky models pay more attention to performance, but at the expense of bulky network models. Compact models included SSR-Net [12] and DenseNet [48], which emphasize a reduced memory footprint, but sacrifice the accuracy of the models. Table 5 reports the *MAE* values on MORPH2, FG-NET, and LAP for the set of state-of-the-art network models for age estimation, including both bulky and compact ones.

Compared with the bulky models, our model not only had a relatively small size, but also achieved lower *MAE*s. This was because we first broke down the complex task of age estimation into three simple subtasks. Then we learned and fused the gender-specific, race-specific, and age-specific features, which provided more instructive age-related information than only learning the age features. Another possible reason was that bulky models often require a large number of samples to train the network; however, the sample of the age-related databases was still insufficient. For example, MORPH, the most popular age dataset, contains only 55,000 face images, which is not enough for the training of the bulky models. Therefore, the lack of samples limited the performance of large-scale models, but these samples were enough to train the compact models to achieve a good performance. Although IMDB-WIKI, the largest age-related face dataset, contains 523,051 face images, it contains too many wrong label samples such as no face or multiface images [10]. Therefore, it was not suitable for training the model directly. Compared with the compact models, our proposed method could make a good tradeoff when both accuracy and efficiency were concerned.

### 4.4. Ablation Analysis

As mentioned above, human aging is a complex process and is easily affected by race and gender. To better learn the complex age features, we first utilized Gender-Net, Race-Net, and Age-Net to learn the gender, race, and age features. Then, we fused these features to form more robust and discriminative features for age estimation. To evaluate the effectiveness of the multifeature learning on age estimation, we conducted the following comparative experiments by removing Gender-Net, or Race-Net, or both of them from our proposed method. Specifically, we took Age-Net (Anet) as the baseline, and we compared Gender-Net (Gnet) and Race-Net (Rnet) as Anet + Gnet, Anet + Rnet, and Anet + Gnet + Rnet (i.e., the proposed method), respectively.

**Figure 5:**
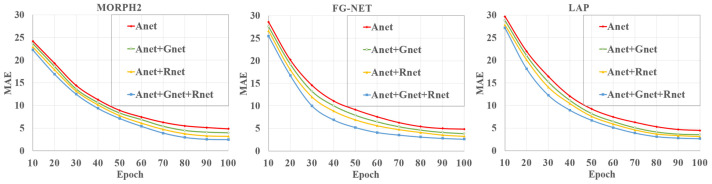
The *MAE*s of the proposed method with different combinations of subnetworks.

**Figure 6:**
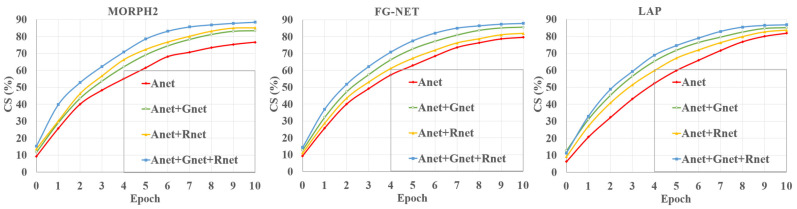
The *CS* of the proposed method with different combinations of subnetworks.

Figure 5 shows the *MAE*s of the proposed methods with different combinations of subnetworks. *CS* is presented to show the performance of different combinations of subnetworks in Figure 6. It clearly shows that Anet+Gnet and Anet+Rnet consistently outperformed the baseline Anet, demonstrating the effectiveness of both the gender- and race-feature-learning procedures. Moreover, Anet+Gnet+Rnet (i.e., the proposed method) yielded the best result. This was because age estimation was significantly affected by race and gender according to the above analysis. For different targets (race, gender, and age), we used different subnetworks to extract different kinds of features and form more robust and discriminative features. This process improved the performance of age estimation and made our proposed method have lower *MAE*s and higher *CS* than the single-feature-based learning module. Therefore, the multifeature learning network could exploit more discriminative and robust age features than a single learning module for age estimation.

**Figure 7:**
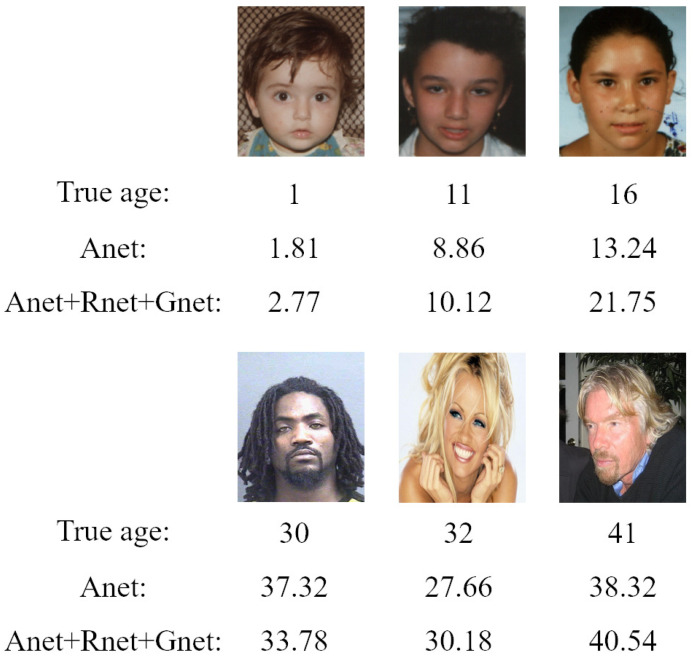
The predicted ages of Anet and Anet+Gnet+Rnet on some samples of different age groups.

To further explore the role of Gnet and Rnet, we conducted experiments on different age groups. Figure 7 presents the prediction results of some samples of different age groups. We can see that the performance of Anet and Anet+Rnet+Gnet was similar when predicting the age of minors. The performance of Anet+Rnet+Gnet was better than single Anet at predicting the age of adults. This was because people of different genders or races have some differences in their aging patterns, and our proposed model could better utilize gender features, which could make the model more flexibility at predicting the age of people of different genders or races. For example, women do not have beards when they are minors or adults, and most men grow a beard in adulthood. Therefore, beards could be used to predict the age of men, but not women.

To validate the effectiveness of our regression-ranking-age estimator, we compared the regression estimator, ranking estimator, and regression-ranking estimator on the age estimation task, respectively. Specifically, for the regression estimator, we removed the ranking estimator and preserved only the regression-age estimator. In contrast, for the ranking estimator, we removed the regression estimator and preserved only the ranking-age estimator.

**Figure 8:**
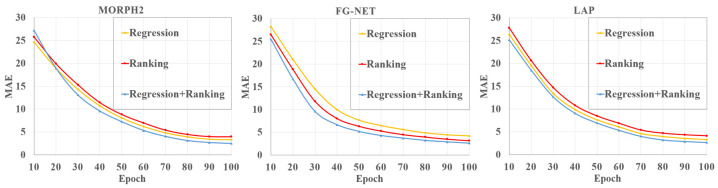
The *MAE*s of the proposed method based on different estimators.

**Figure 9:**
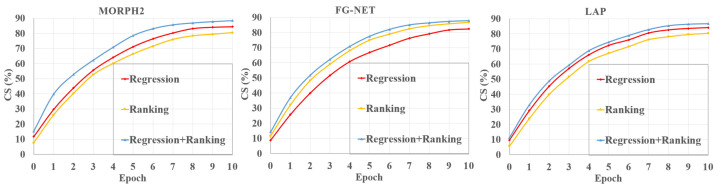
The *CS* of the proposed method with different estimators.

Figure 8 depicts the *MAE*s of the three different estimators on the three databases. *CS* is also presented to show the performance of different age estimators in Figure 9. We can see that our fusion estimator outperformed both the regression-only or ranking-only estimators. This was because our method benefited from not only continuous attributes of age, but also the additional ranking constraints. In addition, our proposed regression-ranking-fusion estimator could make a good tradeoff between the continuity and ordinal relationship of age for the age-feature estimator.

## 5. Conclusions

In this paper, we proposed a compact multifeature-learning method for robust facial age estimation. Specifically, we first used three subnetworks to learn the gender, race, and age features from the same input image, and then, we fused these complementary features to form more discriminative and robust age features. To fully utilize the continuous and orderly property of age labels, we combined the regression and ranking loss to form a regression-ranking estimator for the final age prediction. In addition, our proposed model was very compact with only a 20 MB memory overhead and was suitable to be deployed on devices with limited memory. Experimental results on several benchmark datasets demonstrated that the proposed method achieved a very competitive age-estimation performance compared with the state-of-the-art methods. For future work, it could be an interesting direction to explore more types of age-related information such as working conditions to further improve the age estimation performance, and we will explore ways to make full use of the order and continuity of the age labels, such as using the RMSE loss function, and verify our model on different age groups to better validate the model.

## Figures and Tables

**Table 1:** The basic information and experimental settings of the three databases.

Datasets	Instances	Training (80%)	Testing (20%)	Age Range
MORPH2 [40]	55,000	44,000	11,000	16–77
FG-NET [41]	1002	800	200	0–69
LAP [42]	4691	3612	1079	3–85

**Table 2:** Age distributions of the face images in the MORPH2, FG-NET, and LAP databases.

Age Range	MORPH2	FG-NET	LAP
0–19	7469	710	1246
20–39	31,682	223	4017
40–59	15,649	61	1436
≥60	334	8	310

**Table 3:** The *MAE*s of the different feature-based methods on the MORPH2, FG-NET, and LAP databases.

Methods	Feature	MORPH2	FG-NET	LAP
DEX [1]	Age	2.68	3.09	3.84
MSFCL [6]	Age	2.73	2.71	-
DCP [19]	Age + Race	3.41	3.18	3.32
CNN2ELM [15]	Age + Race + Gender	2.61	2.68	2.72
Ours	Age + Race + Gender	2.47	2.59	2.67

**Table 4:** The *MAE*s of the different estimator methods on the MORPH2, FG-NET, and LAP databases.

Methods	Estimator	MORPH2	FG-NET	LAP
GA-DFL [7]	Ranking	3.25	3.93	3.37
DOEL [22]	Ranking	2.81	3.44	2.93
C3AE [21]	Regression	2.75	2.95	3.05
CNN2ELM [10]	Regression	2.61	2.68	2.72
Ours	Regression + Rank	2.47	2.59	2.67

**Table 5:** The *MAE*s of the different size methods on the MORPH2, FG-NET, and LAP databases.

Methods	Model Size	MORPH2	FG-NET	LAP
DEX [1]	≈500 MB	2.68	3.09	3.84
RankingCNN [23]	≈2.2 GB	2.96	3.96	4.12
SSR-Net [12]	≈1 MB	3.16	4.02	4.17
DenseNet [48]	≈1 MB	5.05	5.68	5.87
Ours	≈20 MB	2.47	2.59	2.67

## Data Availability

No new data were created or analyzed in this study. Data sharing is not applicable to this article.
